# Testosterone serum levels are not predictive of maternal virilization in hyperreactio luteinalis

**DOI:** 10.1007/s00404-020-05745-6

**Published:** 2020-08-19

**Authors:** Mateja Condic, Waltraut M. Merz, Ulrich Gembruch, Dietrich Klingmüller, Birgit Stoffel-Wagner, Ramona Dolscheid-Pommerich

**Affiliations:** 1 Department of Obstetrics and Prenatal Medicine, University Bonn Medical School, Venusberg Campus 1, Building 31, 53127 Bonn, Germany; 2 Division of Endocrinology and Diabetes, Department of Medicine I, University Bonn Medical School, Venusberg Campus 1, 53127 Bonn, Germany; 3 Institute of Clinical Chemistry and Clinical Pharmacology, University Bonn, Venusberg-Campus 1, 53127 Bonn, Germany

**Keywords:** Hyperreactio luteinalis, Hyperandrogenism, Maternal virilization, Pregnancy-associated virilization, Liquid chromatography–tandem mass spectrometry, Electrochemiluminescence immunoassay

## Abstract

**Background:**

Elevated concentrations of circulating testosterone are present in hyperreactio luteinalis (HL), a pregnancy-specific, self-limited condition. HL is associated with maternal virilization in about 30% of cases. The correlation between testosterone levels and maternal virilization has not yet been quantified. Our aim was to identify a testosterone cut-off level which may allow to predict maternal virilization.

**Methods:**

A literature research was performed. Publications were chosen if serum testosterone concentrations and presence or absence of maternal virilization was mentioned. Additionally, we report serial levels of steroids analyzed by Liquid chromatography–tandem mass spectrometry (LC–MS/MS) in one case of HL managed at our institution.

**Results:**

In all, 31 cases fulfilled the search criteria. We found significant overlap between testosterone levels in asymptomatic women and women with signs of virilization (range 6.2–37.3 nmol/l and 13.7–197.5 nmol/l, respectively). The method applied for testosterone analysis was mentioned in three reports only. Peak serum testosterone concentration in our case was 120.3 nmol/l.

**Conclusion:**

From the available data, maternal virilization in HL cannot be predicted by the level of circulating testosterone. However, comparability of results is hampered by the analytical methods applied. LC–MS/MS should preferably be used for reporting concentrations of circulating testosterone.

## Introduction

Maternal virilization during pregnancy or the puerperium is an extremely rare event. It may occur by coincidence, caused by congenital adrenal hyperplasia or adrenal tumors. However, elevated levels of circulating androgens usually result in anovulatory cycles or amenorrhea in affected women. In the vast majority of cases, hyperandrogenemia with or without maternal virilization constitutes a pregnancy-associated condition. Here, excess amounts of testosterone arise from luteomas or hyperreactio luteinalis (HL), where androgens are synthesized in the affected ovaries, and deficiency of placental aromatase, resulting in insufficient metabolization of circulating androgens. Further details are reviewed in Kanova et al. and Hakim et al. [1, 2].

Both, luteomas and HL are benign, self-limited conditions. Luteomas arise from proliferating luteinized cells; some 50% of cases occur unilaterally [3]. Hyperreactio luteinalis can be found in pregnancies with elevated levels of circulating human chorionic gonadotrophin (beta-HCG). Conditions associated with increased HCG production include multiple pregnancy, placental or fetal hydrops, and gestational trophoblastic diseases [4]. However, HL can even occur in pregnancies where beta-HCG concentrations are within normal range. ‘Hypersensitivity’ towards circulating beta-HCG, caused by polymorphisms of the protein or its receptor may result in a gain-of function and may underlie the manifestation of HL with normal beta-HCG levels [5]. Characterized by bilateral multicystic, grossly enlarged ovaries (‘spoke wheel’), symptoms other than maternal virilization may include abdominal discomfort or acute-onset pain caused by ovarian torsion or cyst rupture with the development of hemoperitoneum [6]. The majority of cases, however, either go unnoticed or are a chance finding at the time of obstetric ultrasound or during cesarean delivery [7]. In both conditions, luteoma and HL, hormone levels return to normal range and the ovarian enlargement regresses spontaneously after delivery. A conservative management approach with close follow-up is, therefore, appropriate, reserving surgical intervention for acute complications [8].

Pregnancy-associated virilization may occur in the mother or the fetus, or both. Maternal symptoms include hirsutism in androgen-dependent areas, acne, temporal balding, hypertrophy of the clitoris, and deepening voice, the latter being irreversible in the majority of cases [9]. Female pseudohermaphroditism in the context of pregnancy-associated hyperandrogenemia is exceedingly rare and has been described in less than ten cases; gestational age at exposure may play a role [10].

Maternal virilization in HL occurs in some 30% of cases [11]. The association of circulating testosterone levels with maternal virilization has not been systematically analyzed and predictors are not available. In particular, there is no threshold level of circulating testosterone which could help in risk assessment. This may be due to the following reasons: First, number of reported cases: less than 100 cases are published, the majority being case reports. Second, the analytical method used for measurement.

Unfortunately, there is no standardization for testosterone analysis. Testosterone and its metabolites are usually measured by radio-immunoassays (RIA), immunoassays, or Liquid chromatography–tandem mass spectrometry (LC–MS/MS). Strengths and shortcomings of immunoassays include rapid results and high throughput, but no standardization regarding antibodies used is available [12]. Furthermore, specific laboratory requirements are mandatory for RIAs as radioactive tests. LC–MS allows to analyze multiple steroids within one run with low sample volume; additionally, in contrast to immunoassays, no cross-reactions with other metabolites are observed.

The aim of our study was to correlate maternal testosterone levels in HL with clinical symptoms of maternal virilization. For this, we performed a literature research and compiled the published data. Additionally, we report the clinical course and serial hormonal analyses using LC–MS/MS in one patient under our care who developed maternal virilization during the second trimester of pregnancy.

## Case

**Fig. 1:**
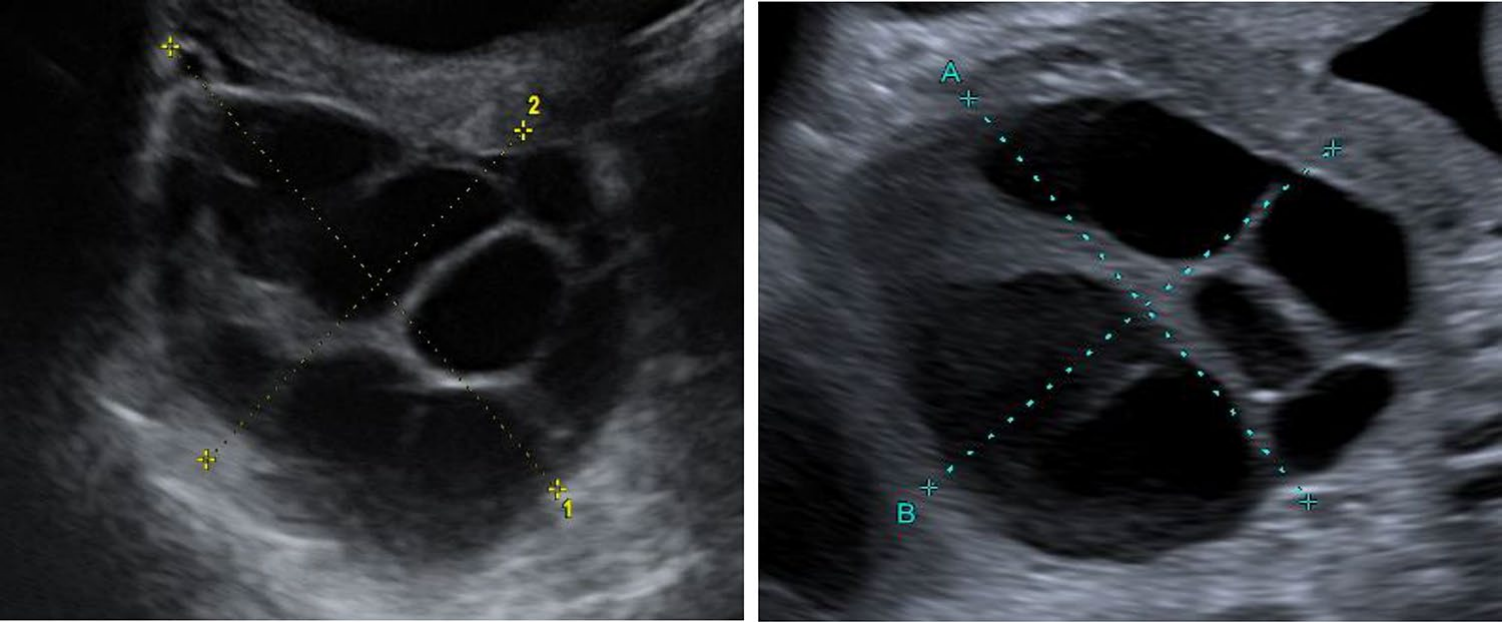
Ultrasound images of the ovaries in a patient with hyperreactio luteinalis at 21 weeks of gestation. **a** Sagittal plane of the enlarged left ovary (67 × 66 mm); **b** sagittal plane of the enlarged right ovary (77 × 60 mm)

A 34-year-old Asian primigravida was referred to our department in the 21st week of gestation. She complained of tachycardia, palpitations, sleeplessness and loss of weight since 4 weeks and had been started on metoprolol (47.5 mg/day) for symptom control. Thyroid function tests had been within normal range on several occasions (see Table 1). She had conceived after ovarian stimulation and insemination. Gestational diabetes had been diagnosed at 17 weeks of gestation. Ultrasound examination revealed an appropriate for gestational age singleton male fetus, and bilaterally enlarged polycystic ovaries (see Fig. 1). Tumor markers (CA125, Inhibin B) and beta-HCG were within normal range. After extensive discussion, a conservative management with close monitoring was agreed upon, and symptoms of ovarian torsion were explained.

**Table 1:** Results of serial hormone analyses during pregnancy and postpartum in a case with hyperreactio luteinalis and maternal virilization

Gestational age (week + day)/days postpartum	21 + 0	31 + 6	32 + 6	35 + 0	36 + 6	Delivery	2 days	5 days	12 days	19 days	27 days
TSH (µU/ml)	0.43	0.86									1.51
fT3 (pg/ml)		2.76									
fT4 (pmol/l)	11.8	12.0									12.7
HCG (mIU/ml)	73009	59131	64228		53166	14227	1977	312	25.8	8.3	4.1
Androstenedione (nmol/l)		49.6			50.4	59.2	67.9	47.1	90.1	29.0	14.3
FSH (mIU/ml)								< 0.30			4.90
LH (U/l)								< 0.30			< 0.30
Progesterone (nmol/l)		543.8	550.1		671.0	69.6	11.3	3.0	< 0.16	< 0.16	< 0.16
ACTH (pmol/l)				9.68	12.5	10.0	5.6	6.2	4.8	5.3	4.6
Cortisol (nmol/l)			469.2		529.9	494.0	378.1	300.8	151.8	220.8	157.3
DHEAS (µmol/l)		1.47	1.4		1.6	2.0	3.2	4.0	2.3	2.3	2.7
Estradiol (pmol/l)		82935.2			93221.4	3171.7	2551.3	1982.3	63.9	< 18.4	< 18.4
FAI (free androgen index)		18.9						28.4	1.9	0.1	
SHBG (nmol/l)		636	655		646	453	403	323	203	161	111
17-OHP (nmol/l)		64.8	46.7		65.1	31.5	22.5	16.0	2.4		2.1
Testosterone (nmol/l)		120.3	112.7				95.0	91.5	3.8	0.2

At 32 weeks of gestation, symptoms of hyperthyroidism had subsided, and metoprolol had been tapered. Ovarian size was stable. She now complained of deepening and hoarseness of her voice; when asked, she confirmed worsening seborrhea and hypertrophy of the clitoris. Hormone analysis revealed excessively elevated androgen levels (see Table 1). A provisional diagnosis of HL was established. The patient was reluctant to undergo any further diagnostic tests. A conservative approach with close clinical and laboratory controls was agreed upon. Further complications did not occur. Induction of labor was performed at 37 weeks of gestation with the aim to reduce prolonged maternal and fetal exposure to high circulating testosterone levels. She delivered a healthy male baby without signs of hyperandrogenism (weight 2920 g, Apgar 9/10/10, umbilical artery pH 7.22).

**Fig. 2:**
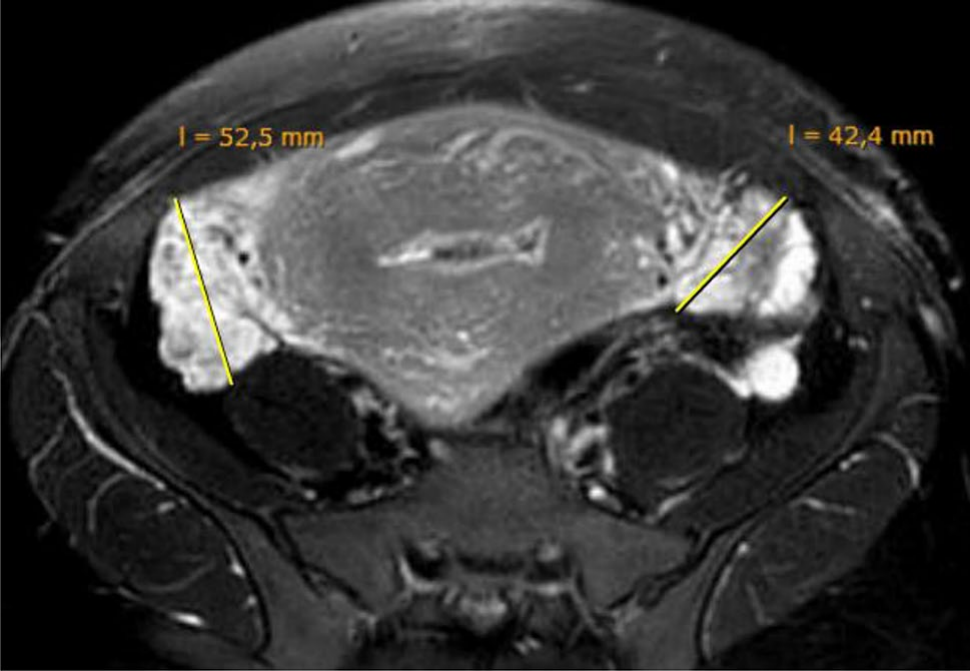
Pelvic magnetic resonance imaging of the same patient, 2 days after delivery. Apart from prominent ovaries (right ovary: 52.5 mm diameter; left ovary: 42.4 mm diameter) no abnormalities were detected

Breastfeeding was initiated successfully without delay. Two days after delivery, magnetic resonance imaging (MRI) was performed to rule out adrenal or ovarian malignancy (see Fig. 2). During the subsequent weeks, serial ultrasound examinations showed a progressive decline of the ovarian volume; serum androgen levels normalized within 20 days. The vocal changes improved gradually; however, 1 year after delivery, a full recovery has not occurred.

## Methods

### Hormone analyses

Venous blood samples were drawn and hormone analyses were performed at the central laboratory of the University Hospital Bonn. Serum steroids (androstenedione, progesterone, cortisol, dehydroepiandrosterone sulfate (DHEAS), estradiol, 17-OH-progesterone and testosterone) were analyzed with LC–MS/MS analysis by MassChrom^®^ assay (Chromsystems, Graefelfing, Germany) according to the manufacturer’s protocol. A Xevo^®^ TQ-S mass spectrometer (Waters, Eschborn, Germany) with electrospray ionization was applied for LC–MS/MS analysis. Adrenocorticotropic hormone (ACTH), luteinizing hormone (LH), follicle-stimulating hormone (FSH), human chorionic gonadotropin (HCG and beta-HCG) and sex hormone-binding globulin (SHBG) were measured by electrochemiluminescence immunoassays (cobas^®^ e801, Roche Diagnostics, Mannheim, Germany).

### Literature research

A literature research was performed covering the time between 1975 and 2018, using PubMed database. The search was restricted to English language. Publications were chosen for the review if androgen concentrations and the presence or absence of maternal virilization were mentioned. Additionally, references of publications were handsearched for further reports.

## Results

**Fig. 3:**
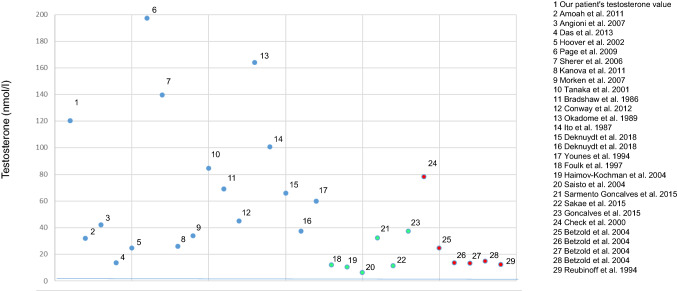
Testosterone serum concentrations and maternal virilization in hyperreactio luteinalis. Blue line: testosterone cut-off level (1.7 nmol/l). Blue dots: cases with maternal virilization; green dots: cases without maternal virilization; red dots: cases without reference to maternal virilization

Our literature research revealed 40 cases with hyperandrogenemia due to HL [1, 5–10, 13–41]. In 29 cases, testosterone levels were measured. Only four publications [10, 13, 15, 18] mentioned the method of testosterone measurement. One case was excluded due to implausibly high values [35]. For further analysis, we focused on the 29 cases where a comparison between testosterone levels was possible, see Fig. 3. In 16 cases, clinical symptoms of maternal virilization occurred. In the other cases, no symptoms were described or maternal virilization was not mentioned in the publication. In clinically asymptomatic women, testosterone concentration varied between 6.2 and 37.3 nmol/l; in cases with maternal virilization, the testosterone concentrations ranged between 13.7 and 197.5 nmol/l, see Fig. 3.

## Discussion

Our analysis of published cases of maternal virilization in hyperreactio luteinalis shows that there is no testosterone cut-off level to predict the development of maternal virilization in HL.

One bias might be the technique used for testosterone measurements. In most of the reported cases, authors did not mention the method used for determination. As immunoassays and LC–MS/MS results are not comparable, a direct comparison between the published cases is difficult. We recommend that future reports on hyperandrogenism should include statements of the laboratory techniques and assays used. Preferably, testosterone values should be obtained with state-of-the-art LC–MS technique due to the absence of cross-reactions. In addition, in contrast to immunoassays, LC–MS offers a large linearity. This is another advantage particularly for cases with high testosterone levels.

Genetic polymorphisms of testosterone receptors may also be a reason for the varying clinical manifestations with the same levels of testosterone [42]. The androgen receptor gene is located on the X chromosome and contains a highly polymorphic region with a variable number of CAG repeats, which normally ranges between 9 and 38 (mean range between 21 and 23) and varies with ethnicity. Epidemiological studies have associated shorter CAG repeats with hyperandrogenic syndromes, and in vitro studies have shown a negative association between androgen receptor activation and CAG repeats length [43].

In all published cases, hormone levels and ovarian size normalized after delivery, obviating the need for surgical interventions during pregnancy, and reserving them for the management of acute complications [35]. Some publications mention delayed lactation due to elevated testosterone levels [24, 26]. In our case, breastfeeding was successfully initiated without delay despite elevated testosterone concentrations (testosterone 95.0 nmol/l on day two after delivery).

Once the testosterone levels return to normal range, most of the clinical symptoms of maternal virilization resolve. However, vocal changes may be irreversible, and without options for medical treatments.

## Conclusions

In conclusion, maternal virilization in the context of HL is a rare, self-limited condition. Interventions during pregnancy are usually not necessary unless surgical complications, ovarian torsion in particular, do occur. Virilization cannot be predicted by the level of circulating testosterone. For the sake of comparability, reports on hyperandrogenemia should state the analytical method used.

## Abbreviations

ACTH: Adrenocorticotropic hormone
DHEAS: Dehydroepiandrosterone sulfate
FAI: Free androgen index
fT3: Free triiodothyronine
fT4: Free thyroxine
FSH: Follicle-stimulating hormone
HCG: Human chorionic gonadotropin
17-OHP: 17-OH-progesterone
SHBG: Sex hormone-binding globulin
TSH: Thyroxine-stimulating hormone
